# Biometric recognition via texture features of eye movement trajectories in a visual searching task

**DOI:** 10.1371/journal.pone.0194475

**Published:** 2018-04-04

**Authors:** Chunyong Li, Jiguo Xue, Cheng Quan, Jingwei Yue, Chenggang Zhang

**Affiliations:** Beijing Institute of Radiation Medicine, State Key Laboratory of Proteomics, Cognitive and Mental Health Research Center, Beijing, P.R. China; Mar Ephraem College of Engineering & Technology, INDIA

## Abstract

Biometric recognition technology based on eye-movement dynamics has been in development for more than ten years. Different visual tasks, feature extraction and feature recognition methods are proposed to improve the performance of eye movement biometric system. However, the correct identification and verification rates, especially in long-term experiments, as well as the effects of visual tasks and eye trackers’ temporal and spatial resolution are still the foremost considerations in eye movement biometrics. With a focus on these issues, we proposed a new visual searching task for eye movement data collection and a new class of eye movement features for biometric recognition. In order to demonstrate the improvement of this visual searching task being used in eye movement biometrics, three other eye movement feature extraction methods were also tested on our eye movement datasets. Compared with the original results, all three methods yielded better results as expected. In addition, the biometric performance of these four feature extraction methods was also compared using the equal error rate (EER) and Rank-1 identification rate (Rank-1 IR), and the texture features introduced in this paper were ultimately shown to offer some advantages with regard to long-term stability and robustness over time and spatial precision. Finally, the results of different combinations of these methods with a score-level fusion method indicated that multi-biometric methods perform better in most cases.

## Introduction

Biometrics is the technology of identifying a person based on the physical or behavioral characteristics of the individual [1]. The physical characteristics, including fingerprints [2], iris [3], eye retina [4], face [5], hand geometry [6], palm-print [7], etc., are associated with the local shape of the body. The feature values of these characteristics are precise and stable for a long time or a lifetime. Therefore, the identification approaches based on these characteristics provide a higher identification rate and have a wider range of applications, such as for security agencies, authentication systems and common-use appliances. Nevertheless, the stability of these features also makes them easier to be forged using modern technological advances [8, 9]. Furthermore, these characteristics cannot ensure that the identified object is a living person. This means that criminals merely need part of the body of the property owner to gain access, leading to a higher probability of damage to the owner. Considering these disadvantages of physical biometrics, it is necessary to develop a second class of biological characteristics based on human behavior, including hand-writing [10], keystroke [11], voice [12], gait [13], etc. These behavioral characteristics include both the behavioral (brain-based) and physical (muscle-based) aspects of a person. Due to the difficulty involved in simulating a person’s brain given the condition of current technologies, it is not easy to imitate this kind of information. On the other hand, it can ensure the identified object is a living person rather than a part of “body”. However, the feature values of this class of characteristics are likely to fluctuate within a certain range rather than be at a precise value, resulting in a relatively lower identification rate. One solution to this problem is to develop a multi-biometric identification technology [14] which can combine these two classes of biometric methods and avoid their disadvantages.

As one of the most important human behaviors, eye movement did not become a behavioral biometric candidate until 2004, although it had played an important role in visual perception research[15–17]. Eye movements, depending largely on neurological control and the extraocular muscle properties of an individual [18], are important human behaviors in interacting with the outside world. There are some advantages behind using eye movement parameters as biometrics. First, the property of a behavioral biometric that combines both physiological and neurological characteristics of an individual makes these parameters impossible to forge. Second, eye movement data collection are more reliable and convenient due to the rapid development of eye tracking technology [19]. Moreover, interaction with portable devices (e.g., Google Glass, laptop, tablets and virtual reality devices) that incorporate eye-based gaze-detection functionalities will be a normal feature in the near future, which will make this biometric approach much more ubiquitous. Third, compared with other behavioral biometrics, eye movement biometrics can be easily combined with other biometric methods—e.g., those using the iris, retina and face—which are also extracted from the face region. Lastly, the capability of unobtrusive data collection makes it a whole process of identification rather than authentication at the outset.

### Related works

Some important works, which have played an important role in eye movement biometric development, are briefly introduced and discussed in the following section.

The viability of eye movement as a biometric indicator was first reported by Kasprowski and Ober [20] in 2004. As the pioneering paper in eye movement biometrics, this work extensively discussed the issue of stimulation selection. As a result, a ‘jumping point’ stimulation was used in the experiment, and first 15 cepstral coefficients were used as identification features. The best result for a database of 9 subjects was achieved using the K-nearest neighbor algorithm, yielding an average false acceptance rate 1.48% and an average false rejection rate of 22.59%.

Kinnunen *et al*. [21] proposed a new person-authentication system using eye movement signals in 2010. It is a task-independent method that can be used in different eye movement biometric experiments regardless of the stimulation being used. The local velocity directions of the gazes, being used as the classification features, were transformed into a discrete probability mass function and were modeled by the GMM-UBM method. An accuracy of approximately 30% EER was achieved, which demonstrates that there is individual information in the eye movements that can be modeled.

An eye movement verification and identification competition has been held three times since 2012. The first competition [22] held in 2012 provided two types of datasets: uncalibrated and calibrated. Most of the competitors treated the eye movement data as a sequence of numbers and used general signal processing and data mining algorithms for feature extraction. The experiment results showed that there might be some unique noise, introduced by a plethora of individual subject-related parameters, in the uncalibrated datasets. As a result, a much higher identification accuracy was achieved with the uncalibrated datasets. Accordingly, equipment calibration is an essential part of eye movement identification. The second competition [23] held in 2014 provided some common basis for eye movement biometrics. The experiment’s results showed that there was no correlation between recognition rate and a sample’s length, the familiarity of an image or the observed image itself. However, significant correlation was found between the rate and subject identification, which may be an influence on better data quality. The competition’s results also showed that the time interval between samples’ recordings had a significant impact on identification rates and that much work needed to be done to make eye movement biometrics easy, fast and reliable. The third competition [24] held in 2015 provided four different datasets to allow the competitors to test their algorithm for different parameters: namely, different visual stimuli and different time intervals between the recordings. The best result, achieved by Anjith George and Prof. Aurobinda Routray, was an overall Rank-1 IR of 95.8%, which was reduced to 70.1% in case of multiple unlabeled recordings per subject. The competition results showed that template aging had a greater effect on recognition accuracy than visual stimulus type. Compared with the identification rates based on the ‘short term’ dataset, a performance loss of 0.4% to 29.4% (M = 13.6%, STD = 10.1%) was observed for the rates based on ‘long term’ datasets. For the top-3 methods, this loss ranged from 9.5% to 29.4% (M = 20.2%, STD = 10.0%). The identification rates based on TEX stimuli were slightly higher than the rates based on RAN stimuli, and the absolute differences ranged from 0% to 18.9% (M = 4.9%, STD = 4.9%).

Komogortsev *et al*. [25] have done a great deal of research on the subject of eye movement biometrics. Complex eye movement pattern biometrics (CEM-B) was first proposed in 2011 [26] and expanded in 2013 [27, 28]. A number of scanpath-based biometric features were extracted from the eye movement records, including fixation count, average fixation duration, average saccade amplitudes, average saccade velocities etc. An EER of 16.5% and Rank-1 identification rate of 82.6% was obtained in a text-reading experiment on account of 32 subjects. The effects of eye tracking specification and stimulus presentation on the biometric viability of this model were also evaluated. The experiment’s results indicated that the eye tracking equipment should be capable of at least 0.5° spatial accuracy and 250 Hz temporal resolution for biometric purposes, whereas stimulus had little effect on the eye movement biometrics. In 2014, they presented a new eye movement biometric model [29, 30] based on a fixation density map (FDM), which is a probabilistic representation of spatial and temporal features related to eye fixations. The best equal error rate of 10.8% and Rank-1 identification rate of 51% were achieved in a dynamic visual stimulus experiment on account of 200 participants. In addition, this model also showed greater robustness than comparable methods in sampling frequencies. Besides, Akram and Marc [31] had also proposed a similar method based on fixation features, saccadic features, pupillary respond features and spatial reading features during reading, and the results they had achieved were an overall accuracy of 95.31% and an average EER of 2.03%.

An eye movement biometric identification method based on low frequency eye movement data was proposed by Andrey and Elena [32, 33] in 2016. A. Rey interwoven lines test was used as stimulus in their experiment, because the eye movement features were computed for the fragments with saccades. Their results had shown that the lowest error rate obtained for the paired comparison algorithm was 15.44% even with the low-frequency eye movement data of 30Hz. Thus, this paper provides a more practical eye movement biometric algorithm compared with those of previous studies.

### Motivation and hypothesis

Although biometric technologies based on eye movement have greatly improved over the last decade, a lot of work still needs to be done to make it more practical. There are three main parts in an eye movement biometric model as follows: data collection, feature extraction and the methods used for feature verification and identification.

The eye tracking equipment and visual stimulus are two important factors in eye movement data collection. The temporal resolution and spatial accuracy of eye tracking equipment determine the quality of eye movement signals. As a result, high-precision eye tracking equipment will contribute to eye movement biometrics. However, due to the restrictions of some special-usage scenarios, high-precision eye movement data cannot always be guaranteed. Therefore, temporal and spatial robustness, which will be analyzed in detail in this paper, are also very important for eye movement biometrics. The visual stimulus is critical for eye movement biometrics because it determines the richness of individual characteristics carried by eye movement trajectories. As a pioneer in the eye movement biometric field, Kasprowski and Ober [20] had performed a detailed analysis on the stimulation selection and has suggested presenting different stimulations each time while also making them as similar as possible in order to both extract the same eye movement characteristics and avoid a learning effect. Based on previous research, the visual task generally falls into two categories: eye-movement restricted visual tasks (e.g., “jumping point” [20], moving balls [34]) and free will visual tasks (e.g., video clips [21], text [26], face image [35]). The eye movement characteristics of eye-movement restricted stimulus mainly represent the properties of the oculomotor plant, because the cognitive strategies from the brain are restricted by the stimulus. In a common free-will visual task, the participant’s reaction time will decrease upon increasing the times for which the participant is watching the same stimulus, which is known as the “learning effect” [36]. On the other hand, the recognition time should be as short as possible for a practical biometric identification method. Thus, the presentation time of the visual stimulus should not be too long. However, in order to get enough multidimensional and idiosyncratic eye movement information, the visual stimulus should not be too simple. Considering all these factors, a well-designed visual search task, which has the same pattern and sufficiently complex content, was applied in eye movement biometric recognition in this paper.

With regard to the feature extraction component, three kinds of features are commonly used in eye-movement biometrics: time series analyses features [37] (e.g., frequency features [20]), fixation- and saccade-related features [26–28] and graph based features (e.g., fixation points on a plane [38] as well as fixation density maps [29]). Different features, representing different characteristics of one’s eye movement, may lead to an eye movement biometric method that differentially performs over a long time interval, suffers from low temporal resolution or has low spatial accuracy—such a finding has not yet been reported. In this paper, we utilized the texture features of eye movement trajectories, extracted with a multi-channel Gabor Wavelet Transform (GWT) method, for biometric recognition, which is a new kind of graph-based eye movement feature. The eye movement trajectory, plotted with raw gaze data, is a representation of the subjects’ information processing results of the specific visual stimulus and can reflect the physiological characteristics of the subject’s oculomotor plant and neurocognitive process to some extent. As a result, there may be some unique characters in the eye movement trajectories. The multichannel Gabor wavelet transform is selected for texture feature extraction due to its spatial locality and orientation-selectivity properties, which are similar to the human visual system [39–42]. Multichannel Gabor wavelets have been widely used in biometric feature recognition, such as face recognition [40, 43, 44] and handwriting identification [45, 46]. An eye movement trajectory is very similar to handwriting in terms of texture features insofar as both have different directional and frequency characteristics for different individuals. Thus, the texture features’ application in eye movement biometrics were explored in this paper, and their performance was compared to three other eye movement features (Local Velocity Direction (LVD) [21], Fixation Density Map (FDM) [29], Complex Eye Movement (CEM) pattern biometric [27]) in various respects.

The feature verification and identification methods, which have been well studied in other biometric studies, will not be analyzed in detail in this paper.

## Methodology

There are three main parts, including data collection, feature extraction and feature verification and identification, in this methodology section. And the protocol (http://dx.doi.org/10.17504/protocols.io.muqc6vw) is also provided, in order to give a clear view of the experiment. This study was approved by the Ethics Committee of Beijing Institute of Radiation Medicine, and all volunteers provided written informed consent to participate in this experiment.

### Data collection

#### Apparatus and software

The eye movement datasets were recorded using a Tobii TX300 eye tracking system running at 300Hz, with vendor-reported spatial accuracy of 0.5°. The stimuli were presented using Tobii Studio software on a 23″, 1920×1080 widescreen monitor at a distance of approximately 600 mm from the subjects. The average validity of 32 participants’ eye movement data was 96.54%±2.36%.

#### Participants

The eye movement datasets employed in this work were collected from a total of 58 subjects (24 males, 34 females) aged 21–33 with an average age of 24.97±2.12. Most of them were the first time to do the visual search task. Before the formal test, they were trained with three groups of the visual search task containing 30 questions in total.

#### Visual stimulus

**Fig 1:**
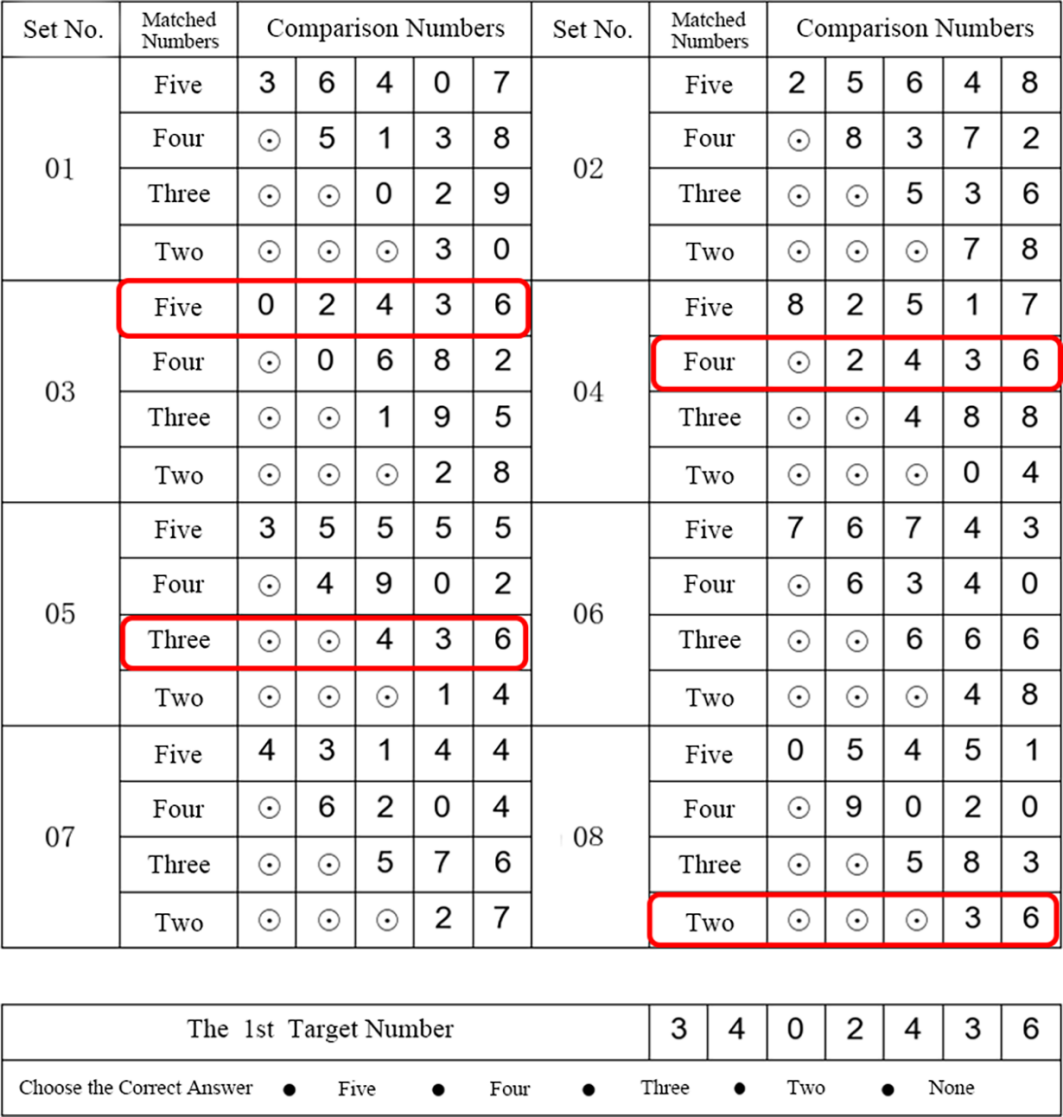
Visual search task. Four comparison numbers (‘02436’, ‘2436’, ‘436’, ‘36’) are matched with the target number (‘3402436’), and the right answer is ‘Five’ which is the length of ‘02436’.

In our eye movement experiment, a novel visual search task (Fig 1, all Chinese characters are converted into English) was designed to improve the accuracy of eye movement biometric recognition. It was a kind of free-will visual search task with a specific mission for the subjects to fulfill. During the experiment, the participants just need to think about how to find the right answer as accurately and quickly as possible rather than to browse without any purpose. The visual search task is a series of number search questions that can be carried out with web pages or pictures. Each question consists of a 7-digit target number and some comparison numbers whose lengths range from 2 to 5. The subjects should compare the target number with the comparison numbers in a right-aligned form to find the longest matched number whose length is the correct answer. That is, there are more than one comparison numbers that are matched with the target number, but only the length of the longest comparison number is the correct answer. As you see in **Fig 1**, there are four Comparison Numbers (‘02436’, ‘2436’, ‘436’, and ‘36’) are matched with the Target Number (‘3402436’), and the Correct Answer is ‘Five’ Which is the length of ‘02436’. Therefore, this visual searching task has 5 different kinds of questions that are balanced during an eye movement data collection experiment. In our experiment, the visual stimuli were presented with Tobii Studio software, and the participants needed to find the correct answer and click on it with the left mouse button.

#### Procedure

Our experiment mainly included two parts: practice and data collection. Before eye movement data collection commenced, every participant practiced on at least 40 questions, which were divided into four groups, to become familiar with the rules of the test. In addition, the learning effect could be reduced when the participants are more familiar with the test.

At the beginning of the data collection part, calibration occurred for every participant in order to get accurate and stable eye movement signals. Each participant’s calibration data was used throughout the experiment. In order to study the effect of long time interval on eye movement biometric identification, the data collection experiment was divided into two trials, and there was a greater than two-week time interval (17.67±3.13d) between them. To collect as much data as possible, there were up to 160 questions in each trial. And these questions were divided into four tests, each consisting of 40 questions.Any participant could take a two minutes’ rest between these tests when they got tired. The questions in these tests were separated by a plus sign, which was designed for the participant to reposition their fixation point. At last 320 eye movement trajectories for each participant (18560 in total) were collected.

### Feature extraction

#### Eye movement trajectories

**Fig 2:**
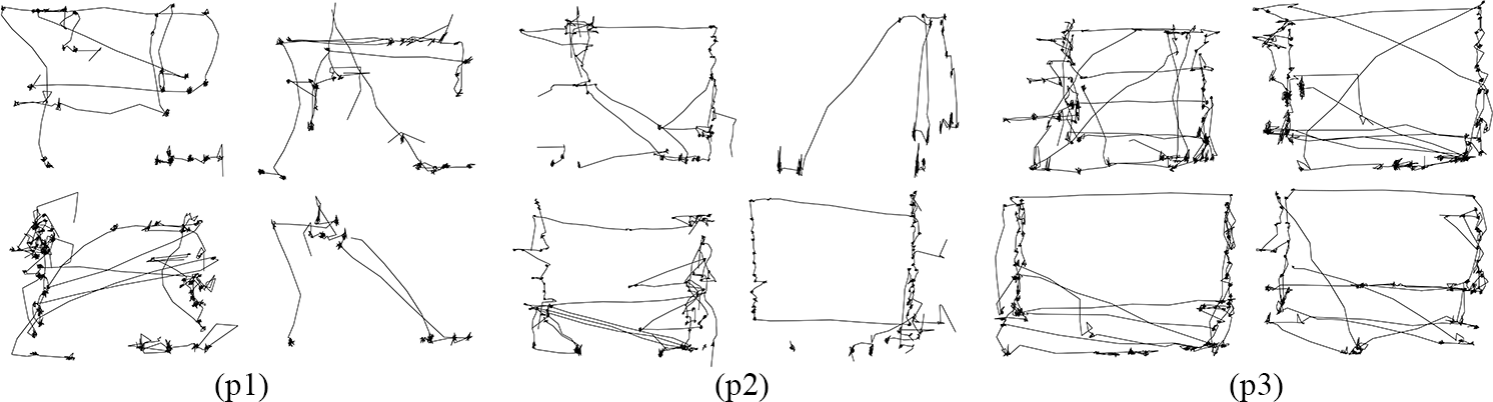
Eye movement trajectories of three different participants.

Compared with fixation data, raw gaze data, recorded in the form of *GazePointX(t)*, *GazePointY(t)* in pixels relative to the screen for each timestamp *t* contains more personal information. As a result, the eye movement trajectories were plotted with raw gaze data rather than fixation data. The image size of eye movement trajectory was set to several fixed values (64×64, 128×128, 256×256 *px*) to test its effect on eye movement biometric identification. Fig 2 illustrates four eye movement trajectories of three different participants.

#### Texture feature extraction

**Fig 3:**
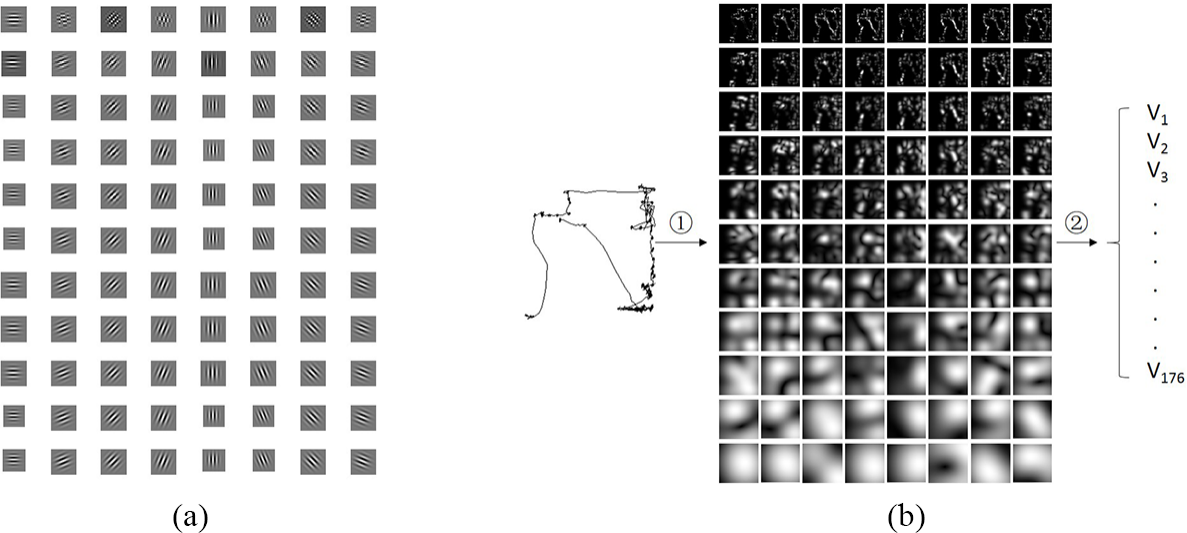
Gabor wavelets and the texture feature extraction process. (a). Gabor wavelets in 11 scales and 8 orientations. (b). ①: Gabor wavelet transform, ②: Calculation of the mean and the variance of texture image’s gray value.

The texture features were extracted from eye movement trajectories with a multi-channel Gabor Wavelet transform as shown in **Fig 3**. Mathematically, the two-dimensional Gabor wavelet is a Gaussian kernel function modulated by a complex sinusoidal plane wave, defined as Eq 1 [47]:

$$\begin{array}{r} G(x,y)=\frac{f^{2}}{\pi \gamma \eta }\exp(-\frac{{x^{\prime }}^{2}+\gamma^{2}{y^{\prime }}^{2}}{2\sigma^{2}})\exp(j2\pi fx^{\prime }+\phi )\\ x^{\prime }=x\cos\theta +y\sin\theta \\ y^{\prime }=-x\sin\theta +y\cos\theta \end{array}$$ (1)

where *f* and *θ* define the radial orientation and frequency of the Gabor kernels, *σ* is the standard deviation of the Gaussian envelops which represents the spatial resolutions of Gabor kernels. In this paper, we use *σ = 1/f*, *γ = 1*, *η = 2*, *ϕ = 0*.

There are only two parameters (*f*, *θ*) need to be designed in a Gabor filter. If the pixel is set as the base unit, the sampling frequency of eye movement trajectory image is 1 Hz. According to the sampling theorem, the highest frequency information contain in the eye movement trajectory image is 0.5 Hz It [48] was shown that for any image of size N×N the important frequency components are limited to f ≥ 4/N Hz. The biggest eye movement trajectory image used in this paper is 256×256 px. Generally, the frequencies are power of $\frac{1}{2}$ and the orientations are $\frac{\pi (j-1)}{4}$, j = 1,2,…,4, whereas the numbers of frequencies and orientations were doubled in this paper to extract more texture information. As a result, the proposed algorithm in this paper employs 88 Gabor wavelets in eleven scales ($f=\frac{1}{2{\sqrt{2}}^{i-1}}$, i = 1,2,…,11) and eight orientations ($\theta =\frac{\pi (j-1)}{8}$, j = 1,2,…,8). This gives a total of 88 texture images for each eye movement trajectory image. The texture features are the mean and the standard deviation calculated from each texture image’s gray value. Therefore, 176 (88×2) features are calculated for each eye movement trajectory image.

#### Feature verification and identification

After extracting features of the eye movement trajectories, we could use any classifiers to solve this pattern recognition problem theoretically, such as weighted Euclidean distance (WED), K nearest-neighbor classifiers (KNN) and support vector machines (SVMs) [49–51], etc. Compared with the other classifiers, SVMs performed better in our preliminary test. As a result, we simply used SVM to identify an eye movement sample in this paper.

Because SVM classifiers are very sensitive to higher-dimension feature vectors, a dimension reduction operation was carried out before the pattern recognition process. Because the training datasets used in this paper are labeled continuous data and some of them are linearly dependent, we just used the linear supervised algorithm for feature dimensionality reduction. First the intrinsic dimension of the 176 texture features is 18 was estimated based on the ‘maximum likelihood’ (ML) algorithm, then dimensionality reduction was accomplished with the ‘linear discriminant analysis’ (LDA) algorithm [52].

For the binary pattern recognition problems, the SVM algorithm seeks to find an optimal separating hyper-plane that defines the largest distance to the nearest point of these two categories [53]. Multi-class pattern recognition problems usually can be solved by constructing decision functions based on combining many binary classification functions [50]. There are two kinds of combination algorithms: namely, one against the rest and one against one. K classifiers are constructed for one-against-the-rest algorithms. The *k*^*th*^ classifier is a hyperplane constructed from class k and the other k-1 class training samples. Then, a majority vote across these K classifiers will be applied to classify a test sample. There are k(k-1)/2 classifiers in total for one against one algorithm, each constructed from two of the K class training samples. Similarly, a majority vote scheme will be applied. In this paper, we select one against one algorithm to solve the identification problem.

In the verification scenario, k-1 classifiers were constructed for each participant. Each classifier was a hyperplane constructed from the feature sample of the specific participant and one other participant. For an unclassified test feature, whether it belonged to this specific participant was determined by the votes of these k-1 classifiers. In the identification scenario, k(k-1)/2 classifiers were constructed for k participants. For an unclassified test feature, the probability it belonged to a certain participant was determined by the votes of these k(k-1)/2 classifiers.

## Results

In the results section, two kinds of datasets were used to demonstrate the biometric results of our method, namely, the short-term dataset (STset) and the long-term interval dataset (LTset). The short-term dataset was obtained by randomly splitting the trial1 data samples into training and testing sets at a specific proportion (70%-30%). The long-term dataset was obtained by using trial1 data samples as a training set and trial2 data samples as a testing set, which were larger sets compared with the STsets. For each participant, 6 test probes and 20 training probes—the mean value of a certain number of feature vectors—were generated from the test and training sets, respectively, using a non-replacement sampling method. As a result, there are (58×6)×58 match scores in total for each of these four eye movement biometric methods. The following results were all averaged over 10 times partition randomly.

### Visual search task evaluation

**Fig 4:**
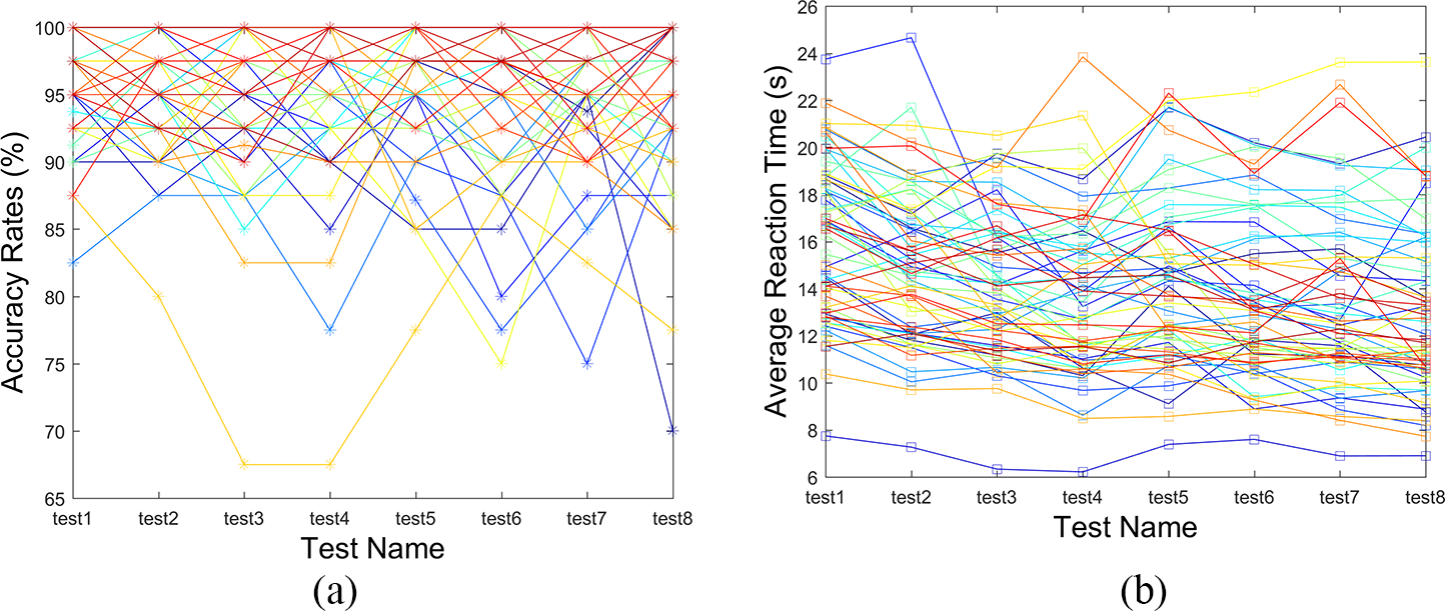
Fifty-eight Participants’ Average Reaction Time (a) and Accuracy Rates (b) in 8 Tests.

The participants’ reaction time has an important influence on the feature values of some eye movement biometric methods (e.g., GFT, FDM and CEM) insofar as these features are closely related to the numbers of fixations and saccades. As shown in Fig 4A, the participants’ reaction times do not fluctuate widely over short intervals or a period of time, indicating that the participants’ proficiencies in this visual search task remain stable after some exercises. This evidence also verifies that our hypothesis about the learning effect of our visual search task was correct, which can greatly contribute to eye movement biometric recognition. In addition, the participants’ accuracy rates (AR) (Fig 4B), which can be used as an objective measure of task performance, were calculated and evaluated in order to eliminate the eye movement trajectories of non-effortful participants. Although some participants’ ARs in certain tests were fairly low compared with other ARs, they were still much higher than the random AR (1/5 = 20%), indicating that these participants’ eye movement data were still valid under a less strict criterion.

### Parameter optimization results

There were two important parameters in our GWT method: namely, the number of eye movement trajectory samples per probe (NoET: 2 4 8 16) and the image pixels of one eye movement trajectory sample (pixET: 64×64, 128×128, 256×256 px) plotted with MATLAB programs.

Although the characteristic values extracted from these trajectories were not all the same but belonged to the same distribution model associated with a particular participant. To increase the identification rate, the feature vectors used in the recognition procedure were the average values of more than one texture feature vector of eye movement trajectories. Because the variance of the sample mean is inversely proportional to the sample number n ($Var(\bar{X})=\frac{\sigma^{2}}{n}$), the vector values will be more stable with the increase of the number of samples contained in one probe. Thus, the biometric method’s performance will be improved with a greater NoET. However, a greater NoET will also increase the time spent on one probe data collection. The pixET has a great influence on how much detailed information a probe image can carry, which will be extracted by Gabor wavelets and used for biometric identification. The bigger the pixET, the more detailed texture information a probe image can carry. In addition, it may have a great influence on the identification rate. As a result, an appropriate combination of these two parameters should be calculated to get a better result in practical applications.

**Fig 5:**
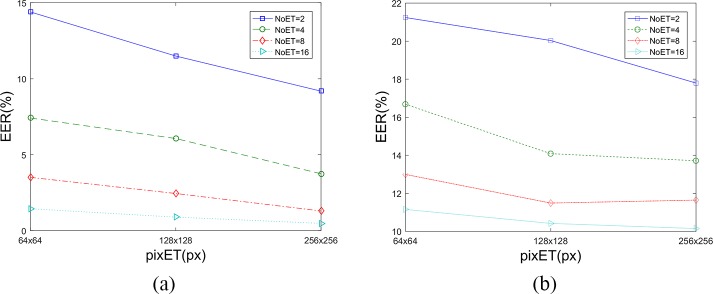
The GWT method’s EERs calculated with different parameter. (a). EER results based on STset, (b). EER results based on LTset.

The EER results (Fig 5) of our GWT method show that the EERs were negatively associated with NoET and pixET in Fig 5A. The EERs were also negatively associated with NoET in Fig 5B but negatively associated with pixET in a certain scope. These results show that the feature vectors become more stable and distinguishable with more eye movement data drawn from one probe image. However, more detailed information was not necessarily better for the LTset data, because some detailed information that was helpful for biometric verification in the STset data was unsustainable, so it could not contribute to biometric verification rates in the LTset data as much as it did in STset. To generate better results in practical applications, pixET should be set to 128×128 and the NoET should be as bigger as possible. However, considering the time requirements and result improvements for a much bigger NoET, the NoET will set to 9 as a trade-off result.

### Verification scenario

The false positive rate (FPR) is defined as the proportion for which an impostor subject is incorrectly accepted by a biometric system. The false negative rate (FNR) is the proportion for which a genuine subject is incorrectly rejected by a biometric system. Finally, the equal error rate (EER) is the proportion at which FPR and FNR are equal. The detection error tradeoff (DET) curve plots FNR on the y-axis against the corresponding FPR on the x-axis. The EER and DET curves are usually used to evaluate the performance of a verification system, which is generally described as a 1-to-1 matching system, because it tries to match the biometrics presented by an individual against the specific biometrics already enrolled.

**Fig 6:**
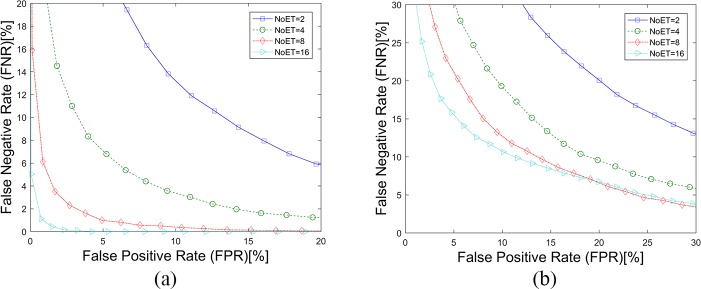
DET Curves of the GWT method. (a). Results based on STset, (b). Results based on LTset.

The detection error tradeoff (DET) curves (Fig 6) plot the results of our GWT method with the parameter pixET set to 128×128, and the corresponding EERs are listed in Table 1. An EER of 0.89%, which is a relatively high value in similar eye movement biometric papers, was achieved on STset with the selected parameters that NoET equals 9 and pixET equals 128×128. However, all the EERs are increased on LTset with percentage of 74.25%, 132.23%, 370.16% and 1074.93% which demonstrate that the time interval has a significant influence on our eye movement biometric results as well.

**Table 1:** EERs and Rank-1 IRs of GWT method.

NoET Dataset	EER (%)				Rank-1 IR (%)			
	2	4	8	16	2	4	8	16
STset	11.49	6.06	2.44	0.89	49.02	71.75	88.16	96.44
LTset	20.03	14.08	11.48	10.41	28.19	42.59	55.57	66.12

### Identification scenario

Unlike the verification systems, an identification system is generally described as a 1 to n matching system, where n is the total number in the database. The Rank-1 IR and CMC curve are usually used as the performance evaluation indexes of identification systems. Rank-k identification rate (IR) is the proportion of instances in which the genuine subject’s match score is found within the top k matches. A cumulative match characteristic (CMC) curve plots rank-k IR on the y-axis against rank k on the x-axis.

**Fig 7:**
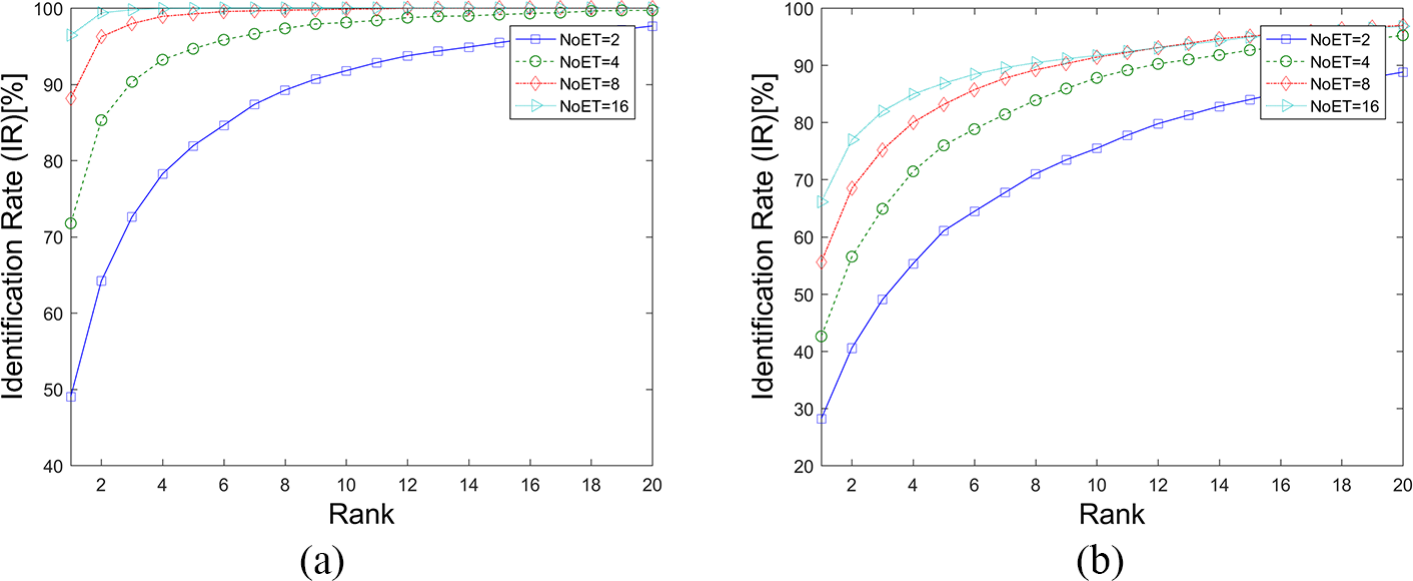
CMC curves of the GWT method. (a). Results based on STset, (b). Results based on LTset.

Fig 7 shows the cumulative match characteristic (CMC) curves of our GWT method based on different datasets (a. STset, b. LTset) with the parameter pixET set to 128×128, and the corresponding rank1-IRs are listed in Table 1. There is a negative correlation between Rank-1 IR and EER—i.e., biometric methods that have smaller EERs always get higher Rank-1 IRs. The Rank-1 IRs of our GWT method were also very good, considering that a random Rank-1 IR is 1.72% (1/58). Compared with the STset Rank-1 IRs, the Rank-1 IR losses of LTset were also very large at 42.50%, 40.65%, 30.96% and 31.44%.

#### Results of other biometric methods

In this section, three other eye movement biometric methods were tested on our datasets. These methods were selected based on their feature extraction methods and suitability for our datasets. The LVD method and FDM method utilize graph-based features for eye movement biometric identification, and the CEM method utilizes fixation- and saccade-based features for eye movement biometric identification. The time series analysis features are commonly used in eye-movement restricted tasks, which were not evaluated in this section. The results were calculated with a different probe size (NoET) and different time interval datasets (STset and LTset).

#### Results of the local velocity direction feature method (LVD method)

This eye movement biometric method was proposed by Kinnunen *et al*. [21] in 2010. As a task-independent person authentication method, it could easily be applied to our datasets. In the original paper, optimization analysis was made to these parameters, which had an important influence on the biometric results, including the feature parameters and the GMM-UBM (Gaussian mixture model universal background model) training parameters. The optimization analysis of this method is not among our major problems in this paper, and we simply adopted their preferences: the histogram bins of local velocity direction were set to 27, the number of Gaussians of the GMM-UBM was set to 16, and the relevance factor r used in creating the user-dependent adapted Gaussian mixture models was set to 16. As one of the feature parameters, the window length, corresponding to NoET in our method, was considered in the results evaluation. The best result achieved in the original paper was an EER of 30% for a count of 17 participants.

**Fig 8:**
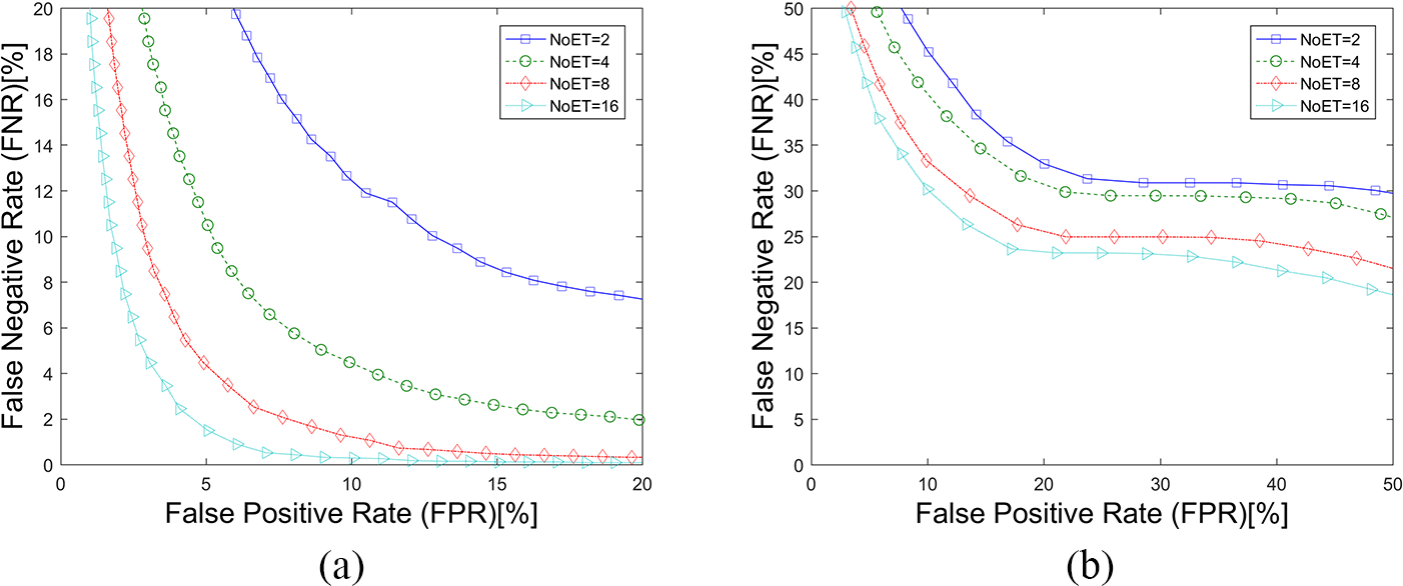
DET Curves for the LVD method. (a). Results based on STset, (b). Results based on LTset.

**Fig 9:**
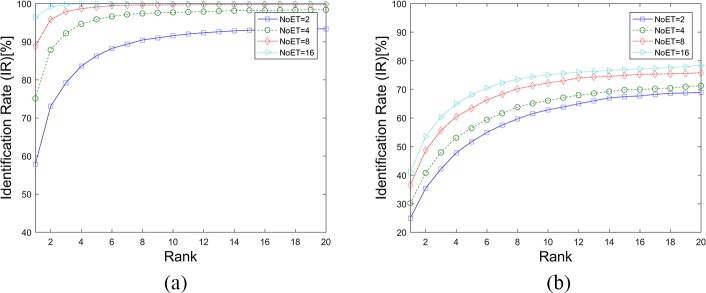
CMC curves for the LVD method. (a). Results based on STset, (b). Results based on LTset.

Fig 8, Fig 9 and Table 2 show the results of the LVD method being applied to our datasets. The performance of this biometric method was also improved upon the increase of NoET. However, when NoET increased to 16, its performance did not improve as significantly as it did when NoET increased to 9. It can be assumed that biometric performance can be improved with an increase of the NoET only before it reaches the limiting value, and improved speed decreases with the increase of NoET.

**Table 2:** EERs and Rank-1 IRs for the LVD method.

NoET Dataset	EER (%)				Rank-1 IR (%)			
	2	4	8	16	2	4	8	16
STset	11.42	6.91	4.73	3.53	57.78	75.11	88.79	96.37
LTset	30.86	29.45	24.94	23.18	24.9713	30.17	36.49	41.03

#### Results of the fixation density map method (FDM method)

This eye movement biometric method was proposed by Komogortsev *et al*. [29, 30] in 2014. This method uses a fixation density map (FDM) as the feature vector of the eye movement during an inspection of dynamic visual stimulus. In the original paper, four different similarity measures (similarity metric (SIM), Pearson’s correlation coefficient (PCC), Kullback-Leibler divergence (KLD)) were used for fixation density map comparisons, and three information fusion methods (simple mean (SM), weighted mean (WM), likelihood ratio (LR)) were used for matching score combinations. The best result was an equal error rate of 10.8% and Rank-1 identification rate of 51% on a large dataset recorded from 200 individuals.

**Fig 10:**
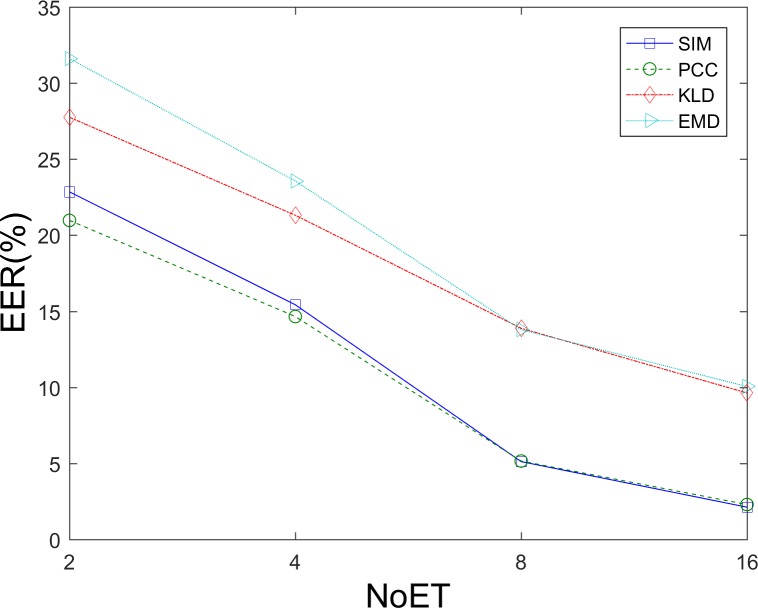
EER Results corresponding to different similarity measures.

**Fig 11:**
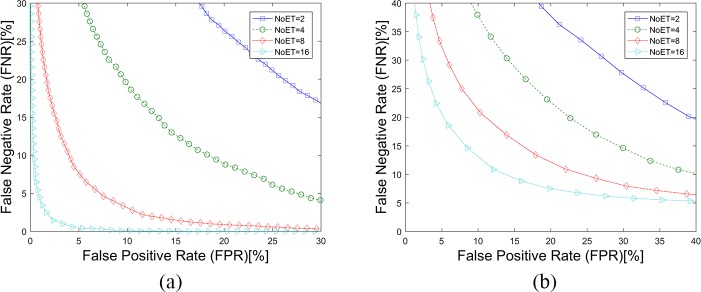
DET curves for the FDM method. (a). Results based on STset, (b). Results based on LTset.

**Fig 12:**
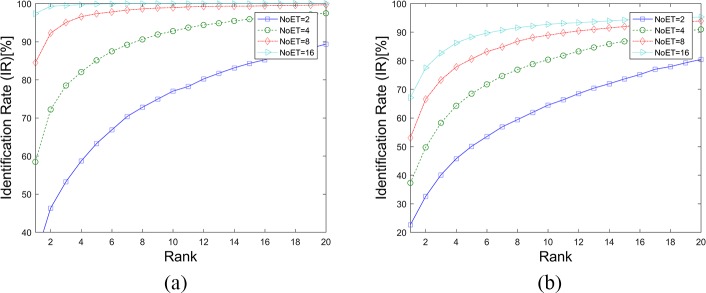
CMC curves for the FDM method. (a). Results based on STset, (b). Results based on LTset.

Fig 10 shows the evaluation results of these four similarity measures of FDM method tested on STsets. In the results presentation and comparison scenario, we simply used the SIM for fixation density map comparisons due to its good performance on our datasets. There was no information fusion problem for the FDM method’s being applied to our datasets, because the fixation density maps of any questions in our datasets were assigned the same weight in matching score combinations. Fig 11, Fig 12 and Table 3 show the results of the FDM method that was applied to our datasets.

**Table 3:** EERs and Rank-1 IRs for the FDM method.

NoET Dataset	EER (%)				Rank-1 IR (%)			
	2	4	8	16	2	4	8	16
STset	23.04	13.86	6.14	1.89	33.30	58.44	84.45	97.35
LTset	28.73	21.33	15.31	11.44	22.58	37.27	53.01	67.06

#### Results of the complex eye movement pattern biometric method (CEM method)

This eye movement biometric method was proposed by Holland and Komogortsev [27, 28] in 2013. For this method, primitive eye movement features based on fixations and saccades are extracted for eye movement biometric identification. Some details of this method have been elaborated upon in the ‘related work’ section. The best result [27] was an equal error rate of 31% and Rank-1 identification rate of 53% on datasets collected with a simple pattern task drawn from 200 subjects. In another paper, the best result [28] was an equal error rate of 16.5% and Rank-1 identification rate of 82.6% based on 32 subjects.

**Fig 13:**
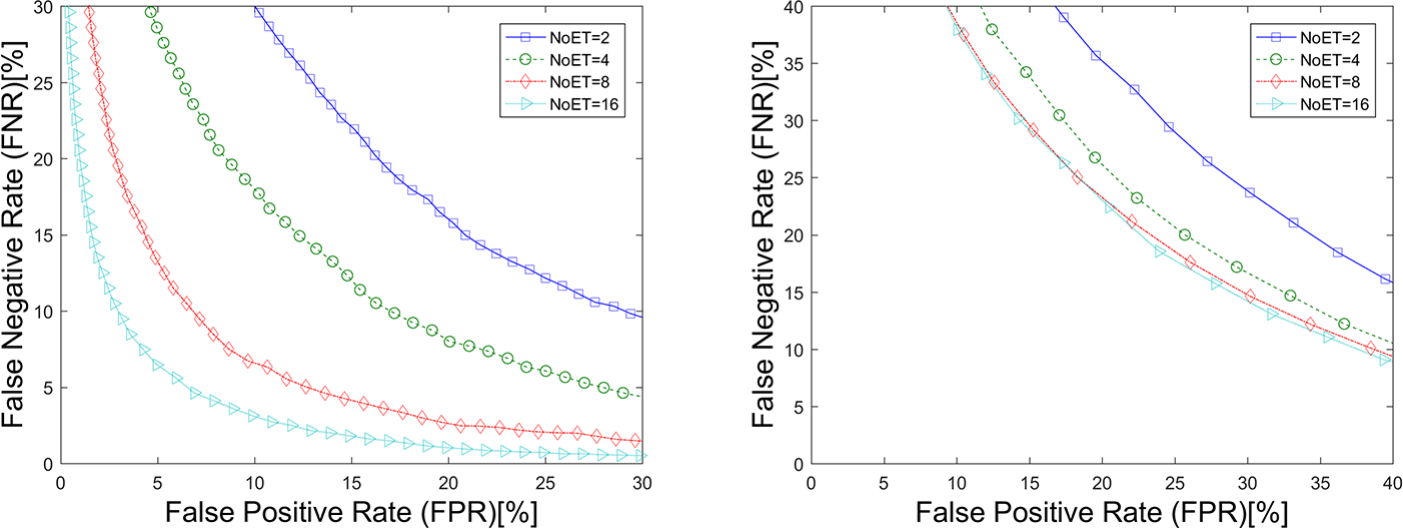
DET Curves for the CEM method. (a). Results based on STset, (b). Results based on LTset.

**Fig 14:**
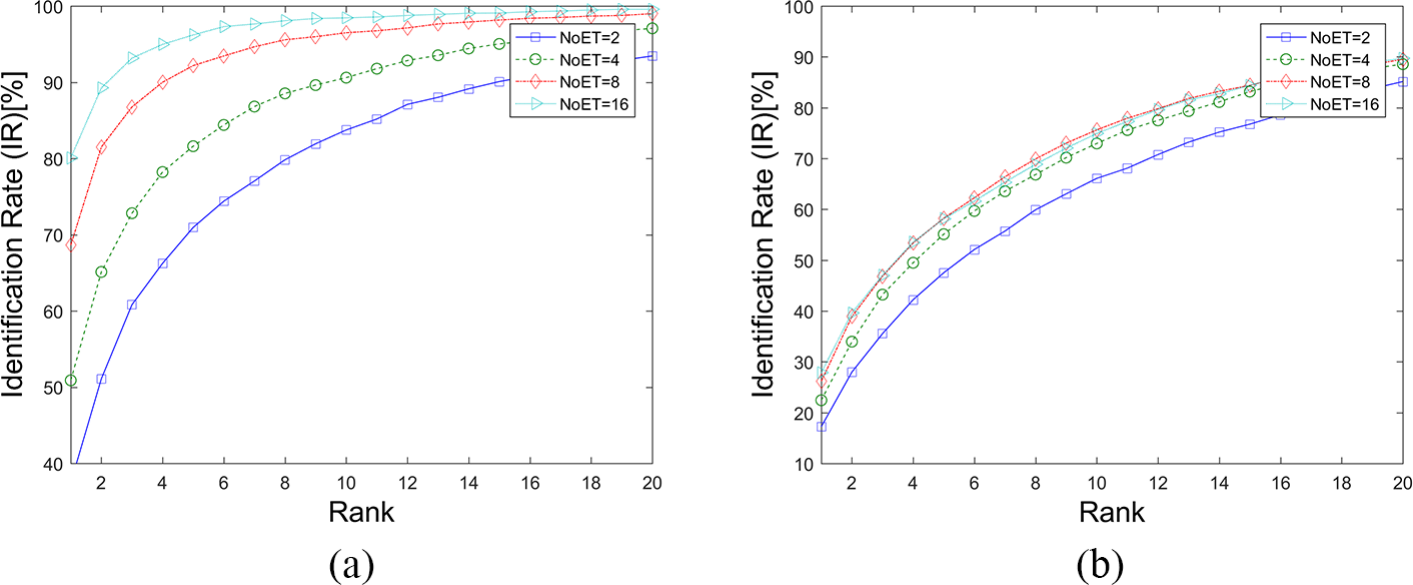
CMC curves for the CEM method. (a). Results based on STset, (b). Results based on LTset.

In this paper, we followed the instructions in the original paper to apply the CEM method to our datasets. The weight vectors of the CEM method were first calculated with an independent test and training data separation. In the STset case, the Trial1 datasets were also randomly separated into test and training datasets with a specific proportion (70%-30%). In the LTset case, the Trial1 and Trial2 datasets were used as training and test dataset, respectively. Then, the weight vectors for these two cases were calculated. Fig 13, Fig 14 and Table 4 show the results of the CEM method as applied to our datasets. The results were better than when compared to the results in the original paper, although they were inferior to the LVD and FDM results.

**Table 4:** EERs and Rank-1 IRs for the CEM method.

NoET Dataset	EER (%)				Rank-1 IR (%)			
	2	4	8	16	2	4	8	16
STset	18.01	13.59	8.16	5.74	37.44	50.89	68.62	80.05
LTset	26.86	22.73	21.63	21.41	17.2414	22.44	26.17	27.87

## Discussion and future research

### The contribution of eye movement trajectories’ gray image to biometric recognition

**Fig 15:**
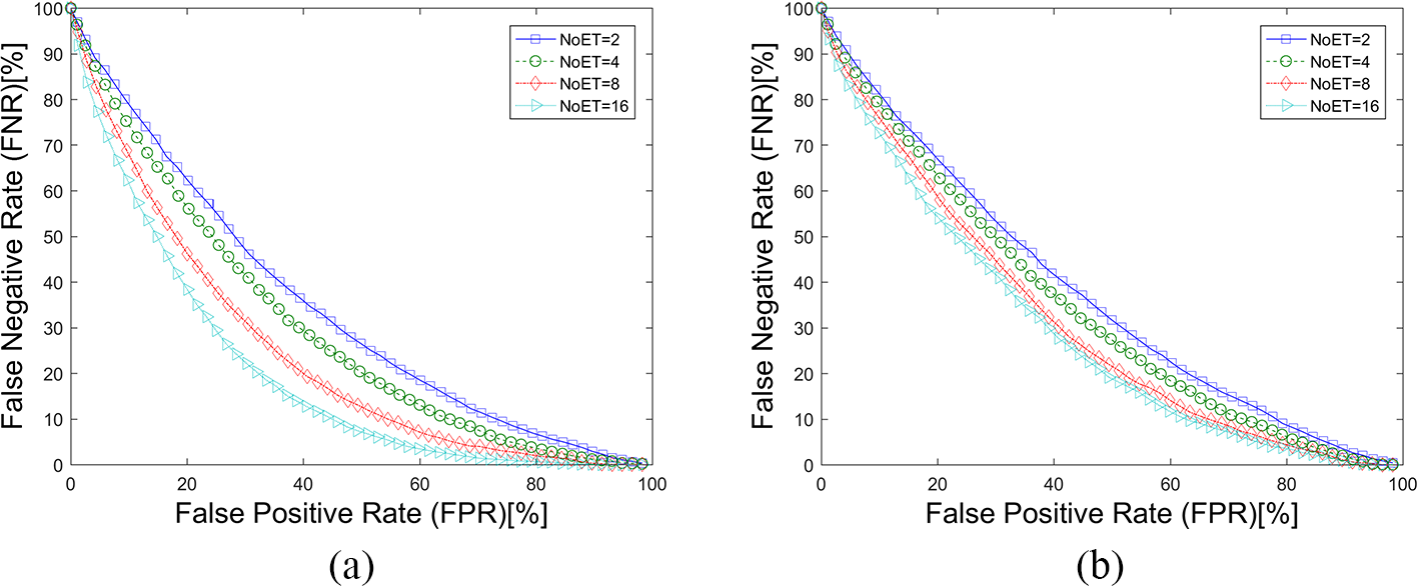
DET Curves for the GI methods. (a). Results based on STset, (b). Results based on LTset.

**Fig 16:**
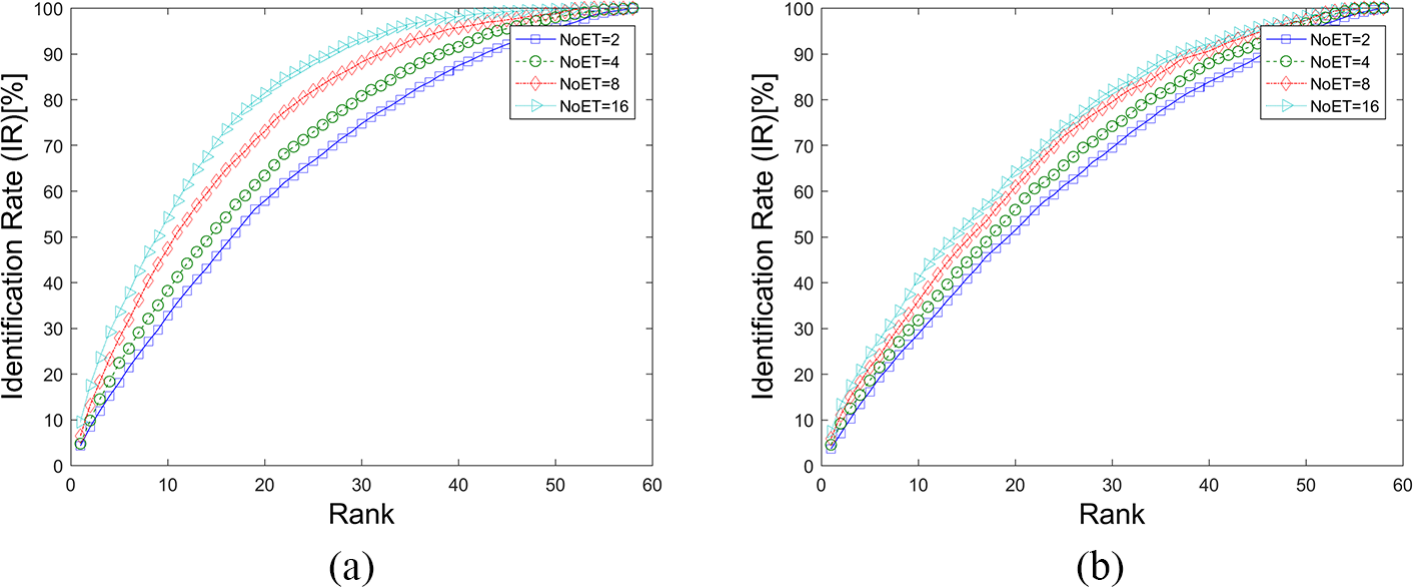
CMC curves for the GI method. (a). Results based on STset, (b). Results based on LTset.

The texture features were extracted from the eye movement trajectories’ gray image (GI). Any device- and person-related noise in the gray image contributes to the biometric recognition results for GWT method. Moreover, the contribution should be evaluated to confirm that the texture features rather than the ‘noise’ is a kind of biological feature. The mean and standard deviation values (GI features) were extracted from the eye movement trajectories’ gray image just as were the GWT features. The SVM classifiers were also used for pattern recognition. Fig 15, Fig 16 and Table 5 illustrate that the GI features had little effect on eye movement biometric recognition, which confirms that texture features are a class of eye movement biometric features.

**Table 5:** EERs and Rank-1 IRs for the GI method.

NoET Dataset	EER (%)				Rank-1 IR (%)			
	2	4	8	16	2	4	8	16
STset	37.93	35.20	30.60	26.61	4.25	4.74	6.43	9.51
LTset	40.83	38.76	36.17	34.85	3.62	4.51	5.86	7.44

### Stimulus’s effects on eye movement biometric viability

Stimulus effects on eye movement biometric viability were evaluated by Holland and Komogortsev in 2013 [27]. The results demonstrate that the stimulus had little effect on biometric viability. However, such a conclusion was drawn from just one class of eye movement features, which might not be applicable to other types of features. Considering the time required for eye movement data collection, this paper’s results reflect the biometric results achieved with NoET set to 9 instead of the best results achieved with NoET set to 16. Table 6 shows the comparison results of these three eye movement biometric methods based on different stimuli. It turned out that the performance of the LVD method was significantly improved. According to the results of the original paper [29], the number of subjects does not influence EER to the extent that it does Rank-1 IR. Therefore, it can be said that the performance of the FDM method is also improved to some degree. Considering the influence played by the number of participants, the biometric results of the CEM method on different stimuli were also significantly improved. In conclusion, the visual searching task proposed in this paper can improve the performance of some eye movement biometric methods compared with ordinary visual tasks.

**Table 6:** Results comparison with same method but a different stimulus.

Methods	Original Paper’s Results	This Paper’s Results
LVD	30% EER on 17 Subs	4.7% EER on 58 Sub
FDM	10.8% EER and 51% Rank-1 IR on 200 Subs	6.1% EER and 84.5% Rank-1 IR on 58 Subs
CEM	31% EER and 53% Rank-1 IR on 200 Subs 16.5% EER and 82% Rank-1 IR on 32 Subs	8.2% EER and 68.6% Rank-1 IR on 58 Subs

### Aging effects on different feature extraction methods for eye movement biometric recognition

Table 7 shows the results of different methods, which were also achieved with NoET set to 9. There are some reports [24] about the effect of template aging on the resulting recognition accuracy. In this paper, the template aging effects on different methods were also evaluated with the difference values of STset results and LTset results. In Table 7, the best are bolded, and the worst results are underlined. To summarize, the GWT method performed best in most cases except the identification scenario on STset. Although we cannot draw a conclusion that the texture features performs best in eye movement biometric recognition, it is enough to prove that the texture features are one of the most promising eye movement biometric features. The DIF values indicate that aging effects have a great impact on all four of these eye movement biometric methods, especially LVD, which is the inherent weakness for all behavior biometric features (e.g., handwriting, keystroke dynamics, gait and voice). However, the GWT method performed best with regard to long-term stability compared with other eye movement biometric features. That is, because it utilizes both macro and specific features, which can be obtained with Gabor wavelet transform of different frequencies, for eye movement biometric recognition.

**Table 7:** Results comparing different methods (DIF being the difference value between the results of STset and LTset).

Methods	EER			Rank-1 IR		
	STset	LTset	DIF	STset	LTset	DIF
GWT	**2.44%**	**11.48%**	**9.04%**	89.16%	**65.70%**	**23.46%**
LVD	4.73%	24.94%	20.21%	91.61%	42.86%	48.75%
FDM	6.15%	15.32%	9.17%	**95.57%**	63.57%	32.00%
CEM	8.17%	21.64%	13.47%	68.62%	26.18%	42.44%

### Temporal robustness and spatial robustness tests

**Fig 17:**
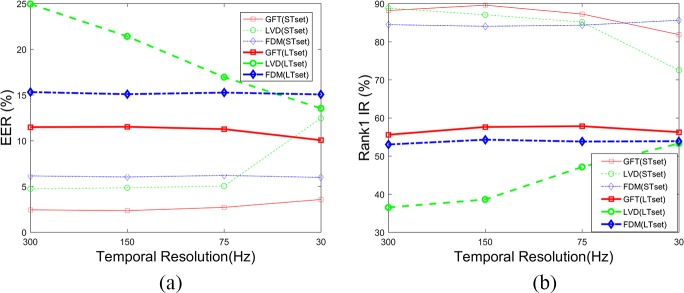
Biometric results of different methods on different temporal resolutions.

The temporal robustness and spatial robustness of the CEM method were tested by Holland and Komogortsev [27], which are not tested in this paper. Their results suggested that eye tracking equipment should capable of at least 0.5° spatial accuracy and 250 Hz temporal resolution for biometric purposes. In this paper, four temporal resolution datasets (300, 150, 75, 30 Hz), which were generated by down-sampling the gaze data, were evaluated. **Fig 17** shows the results of the other three methods on different temporal resolutions. Unlike the CEM method, the GWT method and the FDM method are very robust in temporal resolutions. However, the LVD method’s robustness in temporal resolutions differs a lot on different time intervals. For STset, performance worsens as the sampling frequency decreases in size; for LTset, however, performance improves.

**Fig 18:**
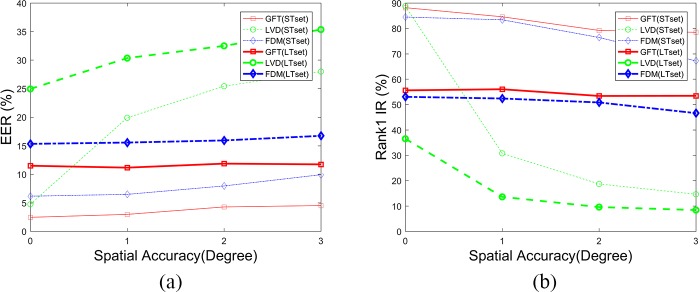
Biometric results of different methods on different spatial accuracies.

Three spatial accuracy datasets (1°, 2°, 3°), which were generated by adding normally distributed random noise to the gaze data, were evaluated in this paper. **Fig 18** shows the results of these three different methods on different spatial accuracy datasets. We can draw the conclusion that the GWT and FDM methods are more stable than the LVD and CEM methods in spatial accuracy.

These results demonstrate that the GWT method proposed in this paper performs just as well as the FDM method does in low temporal and spatial accuracy, and they are both very robust methods. However, both the CEM and LVD methods are very sensitive to temporal and spatial accuracy. That is, because both the filters of fixations and saccades and the local velocity directions need properly high temporal and spatial accuracy. In other words, the biometric methods’ temporal and spatial robustness depends on their feature extraction methods. In addition, the performance improvement of the LVD method on down-sampled LTset shows that less detailed information contained in biometric features contributes to the behavioral biometric method’s long-term stability. The distance between the curves of the same color is also an indicator of long-term stability. Obviously, the long-term stability of the LVD method is significantly improved on down-sampled eye movement data.

### A comparative analysis of score-level fusion results of the four eye movement biometric methods

In general, the biometric features extracted using different methods represent a simplification of personal features contained in the eye movements of different aspects. Theoretically, a fusion method that combines different features will perform better than the best of all these individual method. There are three kinds of fusion methods: namely, feature-level fusion[54], score-level fusion[55] and decision-level fusion. In general, the improvement of these three methods is decreasing. However, the eye movement features extracted with these four algorithms in this paper vary greatly, and therefore they can’t be classified by the same classification method. In this paper, a score-level fusion method was selected. There two steps for a score-level fusion method, i.e., feature standardization and feature fusion. In this paper, Eq 2 was used for feature standardization, and a sum algorithm was used for feature fusion, which is a very simple fusion method for multi-biometric recognition.

$$s^{\prime }=\sqrt{\tanh(\frac{0.01(s-\mu )}{\sigma })+1}$$ (2)

**Fig 19:**
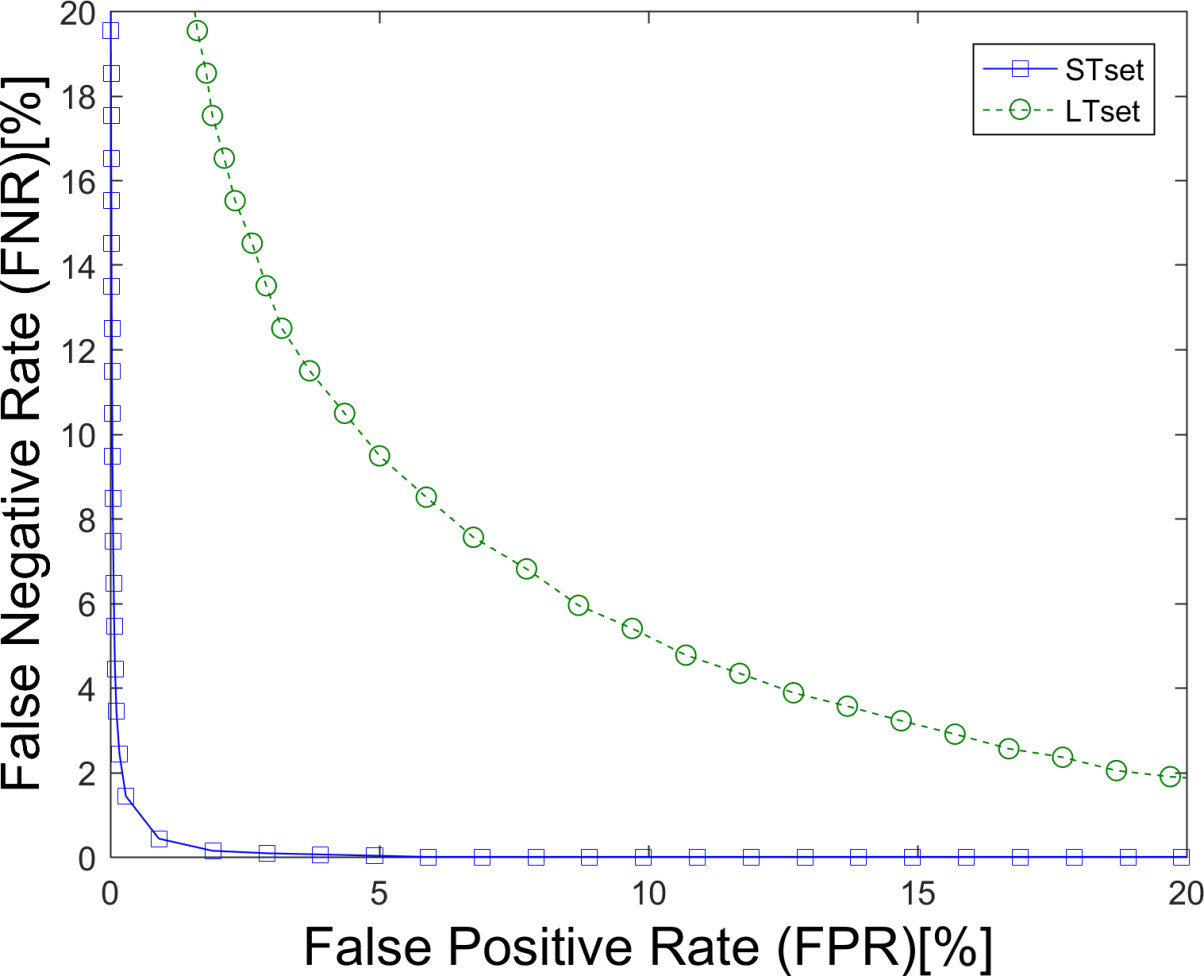
DET curves for the score fusion method of these four features.

**Fig 20:**
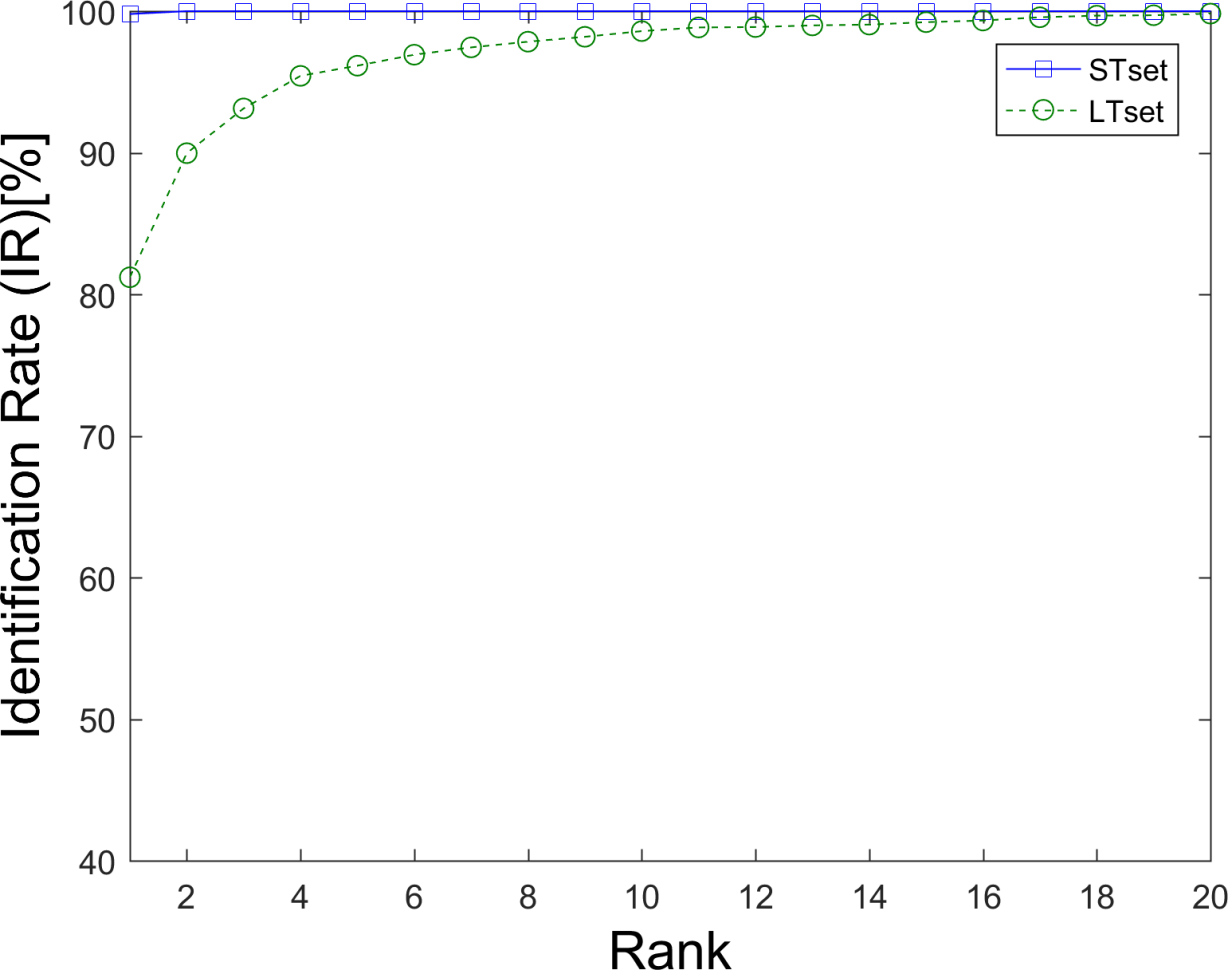
CMC curves for the score fusion method of these four features.

The results in Table 8 show that the biometric performance of fusion methods get better in most cases. The abnormal results, which are bolded in Table 8, often occurs in fusion cases of different classes of features, i.e., graph-based features (GWT and FDM) and fixation- and saccade-based features (LVD and CEM). Moreover, the fusion results of the same class of features often show greater improvement than different classes of features, especially when one of them has relatively lower recognition rates. The best results, which were achieved with a score-level fusion of all four of these methods, were 0.66% EER and 99.79% rank-1 IR on STset data and 7.16% EER and 81.20% rank-1 IR on LTset data. **Fig 19** and **Fig 20** shows the DET and CMC curves of the score-level fusion results of all of these four eye movement biometric methods.

**Table 8:** Score-level fusion results of different eye movement biometric methods.

Fusion Number	Fusion Components	EER (%)		Rank-1 IR (%)	
		ST	DT	ST	DT
1	GWT	2.44	11.48	88.16	55.57
	LVD	4.73	24.94	88.79	36.49
	FDM	6.14	15.31	84.45	53.01
	CEM	8.16	21.63	68.62	26.17
2	GWT+LVD	1.82	**12.93**	97.32	**53.67**
	GWT+FDM	1.56	8.87	96.46	73.44
	GWT+CEM	**3.35**	**11.60**	88.36	**47.29**
	LVD+FDM	1.74	**15.40**	98.56	58.07
	LVD+CEM	2.44	16.49	95.89	53.67
	FDM+CEM	3.40	14.05	91.58	**47.12**
3	GFT+LVD+FDM	0.78	**9.56**	99.71	**70.74**
	GWT+LVD+CEM	1.18	10.12	98.96	62.84
	GWT+FDM+CEM	**1.65**	**9.33**	97.58	**69.77**
	LVD+FDM+CEM	1.10	11.78	99.33	66.81
4	GWT+LVD+FDM+CEM	0.66	7.16	99.79	81.20

### Future research

The visual search task proposed in this paper did improve the performance of the eye movement biometric methods. However, long-term stability remains as the main problem of all these methods, although score-level fusion of these biometric methods can address this problem to a certain degree. Compared with physiological characteristics, behavioral characteristics are more likely to be affected by template aging; such characteristics include many factors, such as fatigue, learning effects and some uncontrollable random factors, which illustrates a kind of inherent disadvantage of behavioral characteristics. To solve this problem, we can try to upgrade the eye movement task, improve the feature extraction methods or try some other biometric fusion methods in the future.

In the comparison results, the new eye movement feature extraction method exhibits some advantages in long-term stability and robustness in temporal and spatial precision. However, the other methods’ parameters were not optimized, and the classification approach to these methods was not taken into account. On the other hand, the feature extraction method proposed in this paper, which is also a kind of task-independent method, has not been tested on other eye movement tasks. These issues require detailed analysis in the future.

The eye movement biometric has been developed for more than ten years. However, most of these research studies were carried out in a laboratory environment with one set of eye tracking equipment. To make it more practical, the effects of eye tracking equipment with different temporal resolutions and spatial accuracies also need to be considered. That is, the eye movement training datasets and eye movement test datasets should be collected with different eye tracking equipment.

Finally, and crucially, on the issue of all behavioral biometric recognition, it is very important to develop a task-independent biometric method. Some methods may perform very well on the same task dataset (i.e., speaking the same words, writing the same characters, typing the same paragraph and walking on the flat floor), but they cannot be applied to different task datasets, which is a significant restriction for real-world deployment. Although there are some task-independent feature extraction methods for eye movement biometric recognition, none of these are task-independent features. This is an important problem in eye movement biometric recognition, which needs to be solved in future research.

## Conclusion

Our research includes designing a new stimulus material and introducing a new class of eye movement features for biometric recognition. The new stimulus, which is a kind of cognitive task, has some clear advantages in eye movement biometrics. Because the participants can complete the task on their own volitions, the eye-movement traces represent not only the physiological characteristics but also the neurological characteristics of the participants. On the other hand, each question in the task has the same form and consists of randomly generated numbers, which can be easily obtained by a program and cause relatively light learning effects for participants. Because the stimulus can be easily generated with a program, it is more suitable for practical application, in which case a person will be identified many times. The biometric performance of some existing methods was significantly improved with this new stimulus. The features extracted using the GWT method rely on different scales and orientations regarding the texture information of eye movement trajectories. An optimization analysis with these parameters has shown some advantages in long-term stability and robustness in time and spatial precision. Moreover, as a task-independent feature-extraction algorithm, it may have a wider range of application and can be combined with other eye movement feature-extraction techniques to increase the overall accuracy of the identification system. The experiment’s results also introduced some principles to improve the eye movement biometric methods’ long-term stability and temporal and spatial accuracy robustness in the future.

## Supporting information

S1 File Feature recognition results for CEM method. (RAR)

S2 File Feature recognition results for FDM method. (RAR)

S3 File Feature recognition results for GTF method. (RAR)

S4 File Feature recognition results for LVD method. (RAR)
